# Socioeconomic Status and Nutritional Status as Predictors of Food Insecurity in Older Adults: A Case Study from Southern Ecuador

**DOI:** 10.3390/ijerph19095469

**Published:** 2022-04-30

**Authors:** Janneth Encalada-Torres, Victoria Abril-Ulloa, Sara Wong, Samantha Alvarado-Romero, Maria Bedoya-Ortega, Lorena Encalada-Torres

**Affiliations:** 1 Research Group on Public Health, Nutrition, and Physical Activity in the Life Cycle, Cuenca 010204, Ecuador; janneth.encalada@ucuenca.edu.ec (J.E.-T.); victoria.abril@ucuenca.edu.ec (V.A.-U.); sara.wong@ucuenca.edu.ec (S.W.); 2 School of Economic and Administrative Sciences, University of Cuenca, Av. 12 de Abril, Central Campus, Cuenca 010204, Ecuador; 3 Nutrition and Dietetics Program, School of Medical Sciences, University of Cuenca, Av. 12 de Abril, Central Campus, Cuenca 010204, Ecuador; 4 Department of Electrical Engineering, Electronics, and Telecommunication, University of Cuenca, Av. 12 de Abril, Central Campus, Cuenca 010204, Ecuador; 5 School of Medical Sciences, University of Cuenca, Av. 12 de Abril, Central Campus, Cuenca 010204, Ecuador; samantha.alvarado@ucuenca.edu.ec (S.A.-R.); elisa.bedoya@ucuenca.edu.ec (M.B.-O.)

**Keywords:** older adults, socioeconomic status, nutritional status, food insecurity

## Abstract

While life expectancy is increasing due to scientific advancement, quality of life in aging depends, among other factors, on the nutritional status and socioeconomic status of older adults. To determine socioeconomic status and its association with nutritional status as a predictor of food insecurity among older adults in southern Ecuador, a cross-sectional study of 188 older adults in urban areas and 212 in rural areas was conducted. Nutritional status, food insecurity, and socioeconomic status were measured. Data were analyzed using SPSS v 15.0 for descriptive statistics and bivariate analysis. Of the older participants, 59% had malnutrition, the majority women, and 24.7% were in poverty. Underweight was associated with low socioeconomic status for adults between 65 and 74 years old (OR = 7.710; CI 95% = 1.691–35.147), while obesity was associated with low socioeconomic status and non-manual labor (OR = 3.048; CI 95% = 1.268–7.326). Over 80% of older adults living in homes without children younger than 18 and at low socioeconomic status had food insecurity. The prevalence of underweight, overweight, and obesity points to widespread nutritional problems, especially in rural areas, that are significantly associated with low socioeconomic status. This demonstrates the need for multidisciplinary programs and government policies that can contribute to reducing food insecurity among the highly vulnerable older population.

## 1. Introduction

Based on evidence from the United Nations Department of Economic and Social Affairs, the worldwide population of older adults (60 years and older) was 962 million in 2017, with two-thirds living in developing countries. By 2050, 79% of the world’s older population is projected to be concentrated in those countries, a trend which includes Latin America and the Caribbean (ELCSA) [1].

Moreover, a study conducted by the World Health Organization (WHO) on the relationship between nutrition and aging found that between 35% and 40% of older adults have protein-energy malnutrition, selective vitamin and/or mineral deficiencies, inadequate water intake, or obesity [2,3]. The nutritional state of older adults in Latin America has also changed significantly, as malnutrition due to overweight and obesity is prevalent in low-income communities [4].

In countries such as Uruguay and Brazil, 35% and 20% of older adults, respectively, were found to have obesity [5]. The WHO stated in their World Report on Ageing and Health that good nutrition in older adults can have a significant impact on their health and wellbeing but that this depends, among other factors, on socioeconomic status [6].

In Ecuador, there is projected to be an increase in the older population from 7% in 2010 to 18% in 2050, which could become unmanageable from different points of view such as the availability of health services, institutional insurance, economic and social inclusion in the country, and food security for this vulnerable group, if public policy is not improved [7,8,9]. This problem intensifies considering that, currently, six in ten older adults belong to the country’s economically inactive population, dependent on family members or the few government programs available. According to general estimates for Ecuador in 2019, Quintile I corresponds to an average income of USD 51 per month, while the wealthiest, in Quintile V, receive USD 637 or more, indicating a significant difference of 367%. This demonstrates clear wealth inequality that has persisted over the years and shapes living conditions, as is reflected in older adults living at different socioeconomic statuses. The economic activities of study participants throughout their lives include agriculture, crafts, and small businesses, such as running small stores, as well as private employment; of those, 59% have health insurance or social security through public and private institutions. According to the Ministry of Economic and Social Inclusion, six in ten older adults belong to Ecuador’s economically inactive population, dependent on families or the State to be able to survive, which results in deep economic insecurity due to reduced work capacity, lack of social security, and limited access to programs that provide income [9].

Furthermore, a huge difference exists between rural and urban older populations since poverty is much higher in rural areas, and people rely on agriculture and livestock for food and sustenance.

Despite some efforts to improve the lives of older adults, the current socioeconomic and nutritional state of Ecuador’s older population is not well known. A better understanding is important for drawing attention to the problems which exist and contributing to the development of effective public policy. Given this context, the objective of this study is to determine the relationship between socioeconomic status and nutritional status among older adults as a predictor of food insecurity.

## 2. Materials and Methods

This study looked at 400 older adults (65 years and older) randomly selected from rural and urban areas in Azuay, a province of Ecuador. To obtain the sample, the following sampling restrictions were carried out using the EPIDAT v3.1 program: population: 55,834 older adults from the urban and rural parishes of Cuenca, with an expected proportion of 13.8 of overweight associated with poverty in older adults [10] with an error of 5% (CI 95%), obtaining a sample of 204 and applying the survey to a total of 400 older adults. The sample was weighted according to the population of the different urban and rural parishes. For the sampling, urban and rural census zones of the province of Azuay considered by the INEC were used, a simple random sampling was used in each parish by census zone, and in each of these by number of older adults weighted by parish. Older adults with psychiatric illnesses, altered states of consciousness, and hearing or cognitive disabilities were excluded. Informed consent was obtained from all subjects involved in the study, and it was conducted according to the guidelines of the Declaration of Helsinki and approved by the Bioethics Committee at the University of Cuenca (Protocol Code: 2018-059EO; date of approval, 6 August 2018)

A cross-sectional study was conducted on 188 older adults from urban areas and 212 older adults from rural areas. Nutritional status was determined using the WHO criteria food insecurity using the Latin American and Caribbean Food Security Scale (ELCSA), which is divided into two sections: the first section includes questions for households with only older adults, categorized as follows: food security = 0 points; medium food insecurity = 1 to 3 points; moderate food insecurity = 4 to 6 points; and severe food insecurity = 7 to 8 points; the second section is for households with older adults and people under 18 years of age, and the ELCSA scale was categorized with the following score: food security = 0 points, medium food insecurity = 1 to 5 points, moderate food insecurity = 6 to 10 points, and insecurity severe eating = 11 to 15 points [11]; and socioeconomic status using the Socioeconomic Status Stratification Questionnaire from Ecuador’s National Institute of Statistics and Census, which includes the following socioeconomic groups: low, medium-low, medium, medium-high, and high socioeconomic status [12].

For nutritional status, weight and height were measured using a precalibrated digital scale and SECA stadiometer. When measured, participants wore light clothing and were barefoot. Body mass index (BMI) was calculated using WHO criteria to determine the nutritional status of participants: underweight ≤ 23.0; normal > 23 to <28; overweight ≥ 28 to <32; and obesity ≥ 32.

In addition, a questionnaire was used to determine residency, occupation, and access to health insurance. Data were tabulated in SPSS version 15.0. Descriptive statistics were used for analysis, including frequencies, percentages, measures of central tendency (mean), and dispersion (standard deviation). Odds ratio (OR) with a confidence interval of 95% was used to look for associations, and a chi-squared test with a value of *p* < 0.05 was used to determine statistical significance. A multivariate logistic regression was performed to obtain the predictive variables of food insecurity in the case of Ecuador.

## 3. Results

The average age of the sample of older participants was 77 years (SD = ±7.7 years), of which 60.2% were women who were mostly married (49.5%) and widowed (31.8%); 66.5% had a primary education; and the most common occupations held throughout participants’ lives were agriculture (26%), housework (20%), crafts and small business (15.3%), and private employment (10.8%). The majority of participants (59%) were insured through the Ecuadorian Institute for Social Security.

Older adults at low and lower-middle socioeconomic status were most prevalent and more located in rural areas, while those in urban areas were predominantly middle socioeconomic status (Table 1).

The number of older adults with underweight, overweight, or obesity was significantly higher than those with normal nutritional status. Older adults with over- or underweight were mostly located in rural areas. The average BMI was 28.12 (SD ± 4.27).

The most underweight older adults were from rural areas and low socioeconomic status, while those with overweight were located in rural areas and lower-middle socioeconomic status. Obesity was present in both geographic areas but more prevalent among older people in urban areas and lower-middle socioeconomic status (Table 2). On the other hand, low socioeconomic status was significantly associated with underweight nutritional status (OR = 2.31; CI 95% = 1.139–4.73 *p* = 0.015).

Underweight nutritional status was related to low socioeconomic status for the following demographic factors: being between 65 and 74 years old (OR = 7.710; CI 95% = 1.691–35.147; *p* = 0.002), not having a partner (OR = 3.042; CI 95% = 0.998–9.268; *p* = 0.042), having a job involving manual labor (OR = 2.842; CI 95% = 0.960–8.416; *p*= 0.050), and with urban residence (OR = 3.067; CI 95% = 0.961–9.784; *p* = 0.048) (Table 3).

Over 80% of older adults living in homes without children younger than 18 and at low socioeconomic statuses had food insecurity. Meanwhile, 75% of older adults at higher socioeconomic statuses and living in homes with children younger than 18 had food security (Table 4).

An association was found between low socioeconomic status and food security in both types of households: only with older adults (OR = 4.323; CI 95% = 2.604–7.172; *p* = 0.000); and when they lived with children under 18 years of age (OR = 3.782; CI 95% = 1.577–9.071; *p* = 0.002), which was statistically significant (Table 5).

The results of the multivariate logistic regression are determined as predictors of food insecurity, both in households only of older adults and households of older adults with children under 18 years of age, having a manual occupation and belonging to the low and medium-low socioeconomic status (Table 6).

## 4. Discussion

Socioeconomic status is one of the factors that influence nutritional status in older adults, particularly those located in rural areas of the Azuay, Ecuador, where there is a high rate of poverty and residents face malnutrition through different means and in the forms of underweight, overweight, and obesity.

This study characterized a sample of participants according to sociodemographic variables, nutritional status, and income quintile in order to identify important factors that significantly influence food security in older adults. The most prevalent socioeconomic statuses were low (Quintile I), lower-middle (Quintile II), and middle (Quintile III), similar to findings from Chavarría Sepúlveda et al. [4] in a 2017 study conducted in Chile, where the predominant socioeconomic quintiles were IV and V, corresponding to the lowest levels.

The research presented here also found that the lowest socioeconomic quintiles were most evident in older adults 65–84 years old, female, married or widowed, with complete or incomplete primary education, and living in rural areas. These findings are comparable to those from studies conducted in Brazil by Damião et al. in 2017 [13] and Pinto de Souza Fernandes et al. in 2016 [14], where the older populations were, for the most part, in the second socioeconomic quartile, which was considered to be a risk factor for malnutrition, together with others factors such as being female and not having a formal education.

On the other hand, the rural population, historically, in Latin American countries such as Ecuador has been related to having a general population located in the lowest socioeconomic quintiles, which has been evidenced once in the results of this research in a vulnerable group such as they are the elderly, which could become a double economic burden for the Ecuadorian State, as it is also an economically inactive group.

This study showed that 20% of older adults from urban areas with obesity were lower-middle socioeconomic status, 32% of older adults from rural areas with overweight were lower-middle socioeconomic status, and 13% of older adults from rural areas with underweight were low socioeconomic status. These results suggest a direct relationship between nutritional status and socioeconomic status in older adults, relevant to the WHO’s statement in its World Report on Ageing and Health [6] that good nutrition in older adults can have a significant impact on their health and wellbeing but that this depends, among other factors, on socioeconomic status. A study conducted in Chile also identified the economic resources of older adults as a determining factor in the variety of a nutritional diet [15], while a study in Peru analyzed the same associated factors and had different results: 10.6% in obesity, 21.7% in overweight, and 26.8% in underweight [10].

Of the older adults studied, 24.7% were in Quintile I, which is the socioeconomic status with the lowest economic income, lower than the national poverty line of 25% as of December 2019 per the National Institute of Statistics and Census [7]. Poverty rates are much higher in rural areas, where many residents rely on agriculture and livestock for their survival, than in urban areas. Comparing the numbers here to those from neighboring Peru, the state of vulnerability in Ecuador’s older population becomes especially evident. Among older adults in Peru (60 years and older), 82.7% are insured, and of those, 60.6% are part of the economically active population involved in commerce, agriculture, fishing, mining, manufacturing, housing, transportation, and education, among others [16]. This suggests they have more economic opportunities than older adults in Ecuador (65 years and older). In Colombia, 36.2% of older adults (60 years and older) were economically active as of 2016, lower than in Ecuador, with 74% of those working out of economic necessity and 26% staying occupied after having retired at 57 years old for women and 62 years old for men [17].

Living in poverty definitively affects the nutritional status of older adults by making it difficult for them to access necessary and nutritious food, a situation worsened by health consequences, which have accumulated throughout other stages of life due to structural inequalities, poor diet, and even multiple births, in the case of women, which can lead to premature death. A study from Chile found that economic resources are a determining factor in the selection and consumption of foods in older adults, such that frequently foods with high nutritional value are replaced by those with a lower cost that will produce more satiety [12]. This change from high to low nutritional value is found to be a risk factor for chronic noncommunicable diseases that, in 2017, were among the leading causes of death worldwide: 12.08% ischemic heart disease, followed by 8.34% diabetes mellitus, 7.72% cerebrovascular disease, and 7.34% hypertensive heart disease [6].

Of the older adults studied here, 59.6% had some form of malnutrition (10.3% underweight, 30.8% overweight, and 18.5% obesity), most commonly in those ages 75 to 84 and female. The highest rate of malnutrition was found in older adults who had conducted manual labor throughout their lives, including agriculture, housework, crafts, and small business. This was particularly the case for women who had done paid work throughout their life in addition to domestic activities, leading to long and exhausting days with little to no recreational activities or physical and emotional rest, while also not receiving adequate nutrition to sustain themselves. For the case of underweight, the results of this study were similar to those from a 2018 study in Chile by Durán et al., who reported a 9% prevalence of underweight in the cities of Santiago and Viña del Mar [15]. On the other hand, the results here on obesity were worse than those found in Spain by Suarez-Gomez et al., where the prevalence of obesity among adults over 65 years old and not institutionalized was estimated to be 30.9% in men and 39.8% in women [3]. In Latin America, the nutritional state of older adults has changed considerably with the growing presence of malnutrition through overweight and obesity, leading to a corresponding increase in chronic noncommunicable diseases (NCDs), characteristic of modern times, in low-income communities [4]. This has been evidenced throughout a variety of studies in countries such as Uruguay, where the percentage of older adults with obesity was 35%, in Brazil, where it was 20% [5], and in Peru, where it was 22.3% [18]. These results demonstrate the direct relationship between low income levels and malnutrition, as is supported by a 2020 systematic review and meta-analysis (OR = 2.69; 95% CI: 2.35–3.08; *p* < 0.001) [19].

The World Health Organization, in a study on nutrition during aging [3], found that 35–40% of older adults worldwide have protein-energy malnutrition, selective vitamin and/or mineral deficiencies, inadequate water intake, or obesity. All of these studies show that the nutritional state of older adults is generally getting worse over time due to, among other factors, lack of access to nutritious and healthy foods in low-income areas, such that purchased or prepared foods are low quality and high in carbohydrates and fats [20,21].

In this study, low socioeconomic status only had a statistically significant association with underweight nutritional status, similar to a 2014 study in Peru by Tarqui et al. [10], which found that underweight was significantly associated (*p* < 0.01) with extreme poverty, defined as a household that cannot afford food expenses or other goods and services. There were no statistically significant associations between malnutrition and low socioeconomic status; however, malnutrition was seen in older adults who are female and single, have a high education level, from urban areas, and low socioeconomic status. A study based on the Ecuadorian National Survey of Employment, Unemployment, and Underemployment [22] evaluated household income and education in relation to access to basic household provisions and found that people with a high education level, living in an urban area, and of white or mestizo ethnicity were more likely to have a high income and access to basic household provisions. In contrast, people working in the private sector, who self-identify as indigenous or of Afro-descendant, or who live in rural areas were less likely. Additionally, a study of noninstitutionalized older adults (65 years and older) in the city of Badajoz, Spain, found that the most common nutritional problem faced was obesity, especially in women and people with a low level of education [3].

A study of 1072 older adults in Brazil [23] found that 32.6% had a monthly income of less than two Brazilian minimum wages (BRL 240, which is equivalent to USD 54.62) and greater risk lifestyles, nutritionally speaking since, of those, 40.1% had a low consumption of fresh fruits and vegetables. A study of 115 older adults in Peru [24] found that more than a third were middle socioeconomic status, and another third were lower-middle. All socioeconomic statuses had similar percentages of overweight and obesity except for low socioeconomic status where the prevalence of overweight (56%) was greater; underweight was only found in the lower-middle and middle socioeconomic statuses.

Food insecurity is associated with poverty for older adults who lack the economic resources to access adequate and nutritious food. Among older adults at low socioeconomic status and in households without children less than 18 years old, more than 80% were found to be food insecure; meanwhile, among those at higher socioeconomic statuses and living in households with children less than 18 years old, 75% were food secure. However, an association was found between low socioeconomic status and food security in both types of households: only with older adults (OR = 4.323; 95% CI = 2.604–7.172; *p* = 0.000) and when they lived with children under 18 years of age (OR = 3.782; 95% CI = 1.577–9.071; *p* = 0.002), which was statistically significant. These results were similar to those found by Tang in the United States since the socioeconomic characteristics were significantly related to food insecurity among older adults (*p* < 0.001) [25]. In addition, the results of the multivariate logistic regression determined in our study for the case of Ecuador, as predictors of food insecurity, belonging to the low and medium-low socioeconomic status and having a manual occupation characteristic of this vulnerable group.

The Food and Agriculture Organization (FAO) of the United Nations states that “food security exists when all people, at all times, have physical and economic access to sufficient, safe and nutritious food that meets their dietary needs and food preferences for an active and healthy life” [26]. In this regard, the situation for older adults in developing countries, such as Ecuador, is far from ideal, as evidenced by existing data on malnutrition, high prevalence of chronic NCDs, limited access to adequate food, and lack of resources. In Portugal, malnutrition among a group of older adults was found to be associated with food insecurity due to lack of economic resources [27]. In a study from Mexico, 21.4% of older adults had limited access to food and did not meet the standards for adequate nutrition, 43.2% of men and women were living in a state of multidimensional poverty, and 10% were in extreme poverty. Likewise, according to the National Survey on Health and Nutrition, 28.2% of Mexican households were classified as having moderate or severe food insecurity, with rural, indigenous, and low socioeconomic status households being the most affected at percentages of 35.4%, 42.2%, and 49.5%, respectively [28]. A study of 118 older adults in Chile [4] found that 56.5% of men and 51.6% of women had a normal nutritional status, which was related to being a professional, having a high level of education, and belonging to Income Quintile V (highest socioeconomic status). Meanwhile, obesity was related to being male, having a partner, and doing intense physical activity; overweight was related to a high school education and Income Quintile III; and an elementary school education was related to Income Quintile II and not being a professional.

In addition, among the limitations of the study were not having investigated biochemical markers of nutritional status due to their high cost in a developing country like ours and the difficult access to consent for taking biological samples by the study group.

## 5. Conclusions

The state of malnutrition in older adults is concerning, especially for those at low socioeconomic status and living in rural areas who lack the economic resources and income to access a nutritious diet. Aging within a context of poverty has significant consequences for the nutritional status of older adults, along with impacting their general quality of life. It is the only epidemiological study in Ecuador that has reported food insecurity in this age group, and the data are alarming since most older adults have some degree of food insecurity. Therefore, it is important and urgent for governmental policies and programs to promote better nutritional status among older adults living in poverty both in Ecuador and in other Latin American and developing countries worldwide. Additionally, interinstitutional support is necessary to build alliances that can adequately ensure the health and wellbeing of older adults.

## Figures and Tables

**Table 1:** Older adults by socioeconomic status and geographic area.

Socioeconomic Status	Geographic Area		
	Urban n = 188 (47%)	Rural n = 212 (53%)	Total n = 400 (100%)
Group D (Low socioeconomic status)	17 (4.2)	82 (20.5)	99 (24.7)
Group C− (Lower-middle socioeconomic status)	51 (12.8)	92 (23.0)	143 (35.8)
Group C+ (Middle socioeconomic status)	64 (16.0)	37 (9.3)	101 (25.3)
Group B (Upper-middle socioeconomic status)	50 (12.45)	1 (0.25)	51 (12.7)
Group A (High socioeconomic status)	6 (1.5)	0 (0.0)	6 (1.5)

**Table 2:** Classification of socioeconomic status of older adults by nutritional status and geographic location.

Socioeconomic Status	Nutritional Status							
	Underweight		Normal		Overweight		Obesity	
	Urban n = 13 (3.25%)	Rural n = 28 (7%)	Urban n = 82 (20.5%)	Rural n = 80 (20%)	Urban n = 55 (13.75%)	Rural n = 68 (17%)	Urban n = 38 (9.5%)	Rural n = 36 (9%)
Low	3 (0.75)	16 (4.0)	8 (2.0)	31 (7.75)	3 (0.75)	27 (6.75)	3 (0.75)	8 (2.0)
Lower-middle	5 (1.25)	8 (2.0)	19 (4.75)	36 (9.0)	14 (3.5)	27 (6.75)	13 (3.25)	21 (5.25)
Middle	4 (1.0)	4 (1.0)	26 (6.5)	13 (3.25)	23 (5.75)	13 (3.25)	11 (2.75)	7 (1.75)
Upper-middle	1 (0.25)	0 (0.0)	24 (6.0)	0 (0.0)	15 (3.75)	1 (0.25)	10 (2.5)	0 (0.0)
High	0 (0.0)	0 (0.0)	5 (1.25)	0 (0.0)	0 (0.0)	0 (0.0)	1 (0.25)	0 (0.0)

**Table 3:** Relationship between underweight and low socioeconomic status in older adults according to sociodemographic variables.

	Low Socioeconomic Status	With Underweight n = 400 (100%)	Without Underweight n = 400 (100%)	OR	CI 95%	*p* Value
Age						
65 to 74 years	Yes	14 (8.7%)	69 (42.9%)	7.710	(1.69–35.147)	0.002
	No	2 (1.2%)	76 (47.2%)			
75 to 84 years	Yes	8 (5.0%)	99 (61.5%)	1.010	(0.290–3.517)	0.987
	No	4 (2.5%)	50 (31.1%)			
≥85 years	Yes	10 (12.8%)	42 (53.8%)	1.825	(0.456–7.305)	0.390
	No	3 (3.8%)	23 (19.5%)			
Sex						
Female	Yes	21 (8.7%)	136 (56.4%)	2.440	(0.885–6.725)	0.077
	No	5 (2.1%)	79 (32.8%)			
Male	Yes	11(6.9%)	74 (46.5%)	2.601	(0.791–8.552)	0.105
	No	4 (2.5%)	70 (44.0%)			
Marital status						
Without a partner	Yes	21(10.8%)	107 (55.2%)	3.042	(0.998–9.268)	0.042
	No	4 (2.1%)	62 (32.0%)			
With a partner	Yes	11 (5.3%)	103 (50.0%)	1.858	(0.622–5.555)	0.261
	No	5 (2.4%)	87 (42.2%)			
Education level						
Low	Yes	31 (9.9%)	197 (6.9%)	2.518	(0.945–6.706)	0.057
	No	5 (1.6%)	80 (25.6%)			
High	Yes	1 (1.1%)	13 (14.9%)	1.327	(0.137–12.844)	0.806
	No	4 (4.6%)	69 (79.6%)			
Residence						
Rural	Yes	24 (11.3%)	150 (70.8%)	1.360	(0.443–4.177)	0.590
	No	4 (1.9%)	34 (16.0%)			
Urban	Yes	8 (4.3%)	60 (31.9%)	3.067	(0.961–9.784)	0.048
	No	5 (2.7%)	115 (61.2%)			
Occupation						
Manual labor	Yes	28 (10.6%)	165 (62.5%)	2.842	(0.960–8.416)	0.050
	No	4 (1.5%)	67 (25.4%)			
No manual labor	Yes	4 (2.9%)	45 (33.1%)	1.458	(0.373–5.703)	0.586
	No	5 (3.7%)	82 (60.3%)			

No association was found between overweight and low socioeconomic status; however, obesity was related to a high education level (OR = 38.13; CI95% = 1.119–12.994; *p* = 0.025) and having a job that does not involve manual labor (OR = 3.048; CI95% = 1.268–7.326 *p* = 0.011).

**Table 4:** Socioeconomic status and food insecurity of older adults by household type.

Food Security in Households of Older Adults	Food Insecurity	Food Security	TOTAL
**Socioeconomic Status**	175 (100%)	129 (100%)	304 (100%)
Group D (Low socioeconomic status)	70 (40)	14 (11)	84 (28)
Group C− (Lower-middle socioeconomic status)	70 (40)	48 (37)	118 (39)
Group C+ (Middle socioeconomic status)	27 (15)	39 (30)	66 (22)
Group B (Upper-middle socioeconomic status)	8 (5)	23 (18)	31 (10)
Group A (High socioeconomic status)	0 (0)	5 (4)	5 (2)
**Food Security in Households of Older Adults with children less than 18 years old**			
**Socioeconomic status**	52 (100%)	44 (100%)	96 (100%)
Group D (Low socioeconomic status)	15 (29)	0 (0)	15 (16)
Group C− (Lower-middle socioeconomic status)	14 (27)	11 (25)	25 (26)
Group C+ (Middle socioeconomic status)	16 (31)	19 (43)	35 (36)
Group B (Upper-middle socioeconomic status)	7 (13)	13 (30)	20 (21)
Group A (High socioeconomic status)	0 (0)	2 (2)	1 (1)

**Table 5:** Relationship between socioeconomic status and food security in older adults.

Food Security Households with Only Older Adults						
	Unsafety	Security	Total			
Socioeconomic status	n = 175 (100%)	n = 129 (100%)	n = 304 (100%)	OR	CI 95%	*p* value
Low and medium-low	140 (80)	62 (48)	202 (66)	4.323	2.604–7.172	0.00
Medium, medium-high, and high	35 (20)	67 (52)	102 (34)			
**Food Security Households of Older Adults with Children Under 18 Years of Age**						
	**Unsafety**	**Security**	**Total**			
Socioeconomic status	n = 52 (100)	n = 44 (100%)	n = 96 (100%)	OR	CI 95%	*p* value
Low and medium-low	29 (56)	11 (25)	40 (42)	3.782	1.577–9.071	0.002
Medium, medium-high, and high	23 (44)	33 (75)	56 (58)			

**Table 6:** Multivariate logistic regression.

Parameter Estimates								
Food security in households with only older adults								
	B	E.T.	Wald	gl	Sig.	Exp(B)	95% CI for Exp(B)	
							Lower limit	Upper limit
Intersection	3.463	1.502	5.315	1.000	0.021			
Age	−0.337	0.194	3.023	1.000	0.082	0.714	0.489	1.044
Sex	−0.282	0.288	0.959	1.000	0.327	0.754	0.429	1.326
Having a partner	0.033	0.285	0.013	1.000	0.909	1.033	0.591	1.805
−0.355	0.399	0.792	1.000	0.374	0.701	0.321	1.532
Manual occupancy	−0.703	0.317	4.925	1.000	0.026	0.495	0.266	0.921
Socioeconomic status	−0.844	0.329	6.587	1.000	0.010	0.430	0.226	0.819
BMI_Malnutrition	1.089	1.127	0.934	1.000	0.334	2.971	0.326	27.048
Rural residence	−0.522	0.290	3.245	1.000	0.072	0.594	0.337	1.047
Food security households of older adults with children under 18 years of age								
	B	E.T.	Wald	gl	Sig.	Exp(B)	95% CI for Exp(B)	
							Lower limit	Upper limit
Intersection	−12.210	1.786	46.719	1.000	0.000			
Age	−0.453	0.334	1.843	1.000	0.175	0.636	0.330	1.223
Sex	1.190	0.637	3.488	1.000	0.062	3.288	0.943	11.468
Having a partner	0.575	0.615	0.874	1.000	0.350	1.777	0.533	5.928
Level of instruction	−0.827	0.686	1.455	1.000	0.228	0.437	0.114	1.677
Manual occupancy	−1.639	0.621	6.964	1.000	0.008	0.194	0.057	0.656
Socioeconomic status	−1.085	0.598	3.291	1.000	0.070	0.338	0.105	1.091
BMI_Malnutrition	15.374	0.000	.	1.000	.	4,750,602.457	4,750,602.457	4,750,602.457
Rural residence	0.064	0.572	0.013	1.000	0.910	1.067	0.348	3.271
The reference category is: security.								

## Data Availability

Due to the ethical and bioethical regulations of the University of Cuenca in relation to the people who were part of the study, the database cannot be socialized.
